# A silent outbreak of vancomycin-resistant *Enterococcus faecium* in a neonatal intensive care unit

**DOI:** 10.1186/s13756-020-00755-0

**Published:** 2020-06-16

**Authors:** Ronella Marom, Dror Mandel, Alon Haham, Irit Berger, Amit Ovental, Craig Raskind, Galia Grisaru-Soen, Amos Adler, Jonathan Lellouche, David Schwartz, Yehuda Carmeli, Vered Schechner

**Affiliations:** 1 Department of Neonatology, Lis Maternity Hospital, Tel Aviv Sourasky Medical Center, Weizmann Street, 6423906 Tel-Aviv, Israel; 2 Sackler Faculty of Medicine, Tel Aviv University, Tel Aviv, Israel; 3 Department of Pediatric Infectious Disease Unit, Dana Dwek Children;s Hospital, Tel Aviv Sourasky Medical Center, Tel Aviv, Israel; 4 Department of Clinical Microbiology Laboratory, Tel Aviv Sourasky Medical Center, Tel Aviv, Israel; 5 National Laboratory for Antibiotic Resistance and Investigation of Outbreaks in Medical Institutions, National Institute for Antibiotic Resistance and Infection Control, Ministry of Health, Tel Aviv, Israel; 6 Department of Epidemiology and Preventive Medicine, Tel Aviv Sourasky Medical Center, Tel Aviv, Israel

**Keywords:** Vancomycin-resistant *Enterococcus faecium*, Neonatal intensive care unit, Neonate, Infection, Screening, Outbreak

## Abstract

**Objective:**

To describe the containment of a widespread silent outbreak of vancomycin-resistant *Enterococcus faecium* (VRE-fm) in the Tel-Aviv Medical Center (TASMC) neonatal intensive care unit (NICU).

**Methods:**

Setting - an NICU, participants - 49 cases of VRE-fm-colonized neonatal inpatients.

**Results:**

A newborn was transferred from the TASMC NICU to another hospital and screened positive for VRE-fm upon arrival. All TASMC NICU patients were then immediately screened for VRE and 21/38 newborns were identified as VRE carriers. Interventional measures were strictly enforced. By the end of the outbreak, 49 cases of VRE carriage had been identified. There were no VRE clinical infections. The source of the outbreak was not identified.

**Conclusion:**

Our study highlights the importance of screening implementation in a NICU setting since this outbreak could have been prevented by active screening of all out-born transfer patients and by having adopted mandatory screening into the NICU’s routine procedures. Screening for multi-drug resistant organisms upon admission of all transferred patients to the NICU has been implemented.

## Introduction

Vancomycin-resistant *Enterococcus faecium* (VRE-fm) was first reported in 1988, a time point when it had rarely been isolated in neonates. Its prevalence has significantly increased globally during the following decades [1, 2]. Outbreaks in hospital neonatal intensive-care units (NICUs) have been described [3], and a steady increase in severe VRE-fm infections has been widely reported [3–12]. The emergence of clinical infection is mostly associated with high colonization pressure, as well as with risk factors, most prominent among which are: young age, use of invasive devices, antimicrobial drug administration, immunosuppression, low birth weight, and underlying malignancy [12, 13].

With the increase in the worldwide prevalence of VRE, and considering that neonates are at an increased risk for progressing from silent carriage to active disease, there is a need for effective methodology for the management of outbreaks, specifically within NICUs. The NICU can also serve as a test-case for management of outbreaks, since the NICU’s closed environment provides a unique setting for outbreak control [14]. In Israel, there are no national guidelines for routine screening of newborns for multi-drug resistant organisms (MDROs) in the NICU, and each hospital sets its own policy.

This article describes an extensive silent outbreak of colonization with a newly emergent strain of VRE-fm that was terminated by combined measures comprised of the institution contact isolation, cohorting and enhanced environment cleaning. It is hypothesize that this outbreak could have been prevented by active screening of all out-born transfer patients and by having adopted mandatory screening into the NICU’s routine procedures.

## Materials and methods

### Setting

The Tel Aviv Sourasky Medical Center (TASMC) NICU consists of a total of 49 beds, of which 22 beds comprise a level-IIIB NICU and 27 beds comprise a level-IIA NICU. In addition, there is a special-care nursery with the capacity of 13 beds which is staffed and administered in a facility separate from the NICU. Sick or at-risk neonates are admitted directly after birth into either the NICU or the special-care nursery, depending upon the severity of their illness. They are moved between units as their needs change.

### Patients

The index case was a newborn that needed cardiac surgery and who was identified by positive screening for MDROs upon arrival to another hospital’s NICU.

### Outbreak management and intervention

After the first case of VRE-fm infection was detected, all of the hospitalized neonates were enrolled in active surveillance for VRE colonization according to the following steps:

1. All neonates in the TASMC NICU were immediately screened for VRE-fm.
2. The hospital management and the Ministry of Health (MoH) were informed of a VRE outbreak.
3. A designated VRE group was isolated in the NICU. Since over half of the infants screened positive for VRE-fm, after consulting with the MoH, it was decided that positive and exposed infants (i.e., all infants in the NICU at this time point) will comprise a single cohort and be treated according to the same protocol. This group was separated from all new NICU admissions. The movement of neonates within and between units was restricted, and the entrance of outside staff into the units was kept to a minimum.
4. One room was designated for new NICU admissions and defined as “the clean area”. This room was meticulously cleaned prior to the first new admission. Disposable supplies were either destroyed or moved to the VRE area.
5. Physicians, nurses and nurses’ aides were assigned to work exclusively either in the designated VRE area or in the clean area.
6. A special protocol was followed for cases requiring a specific physician to treat a patient from the VRE group. The protocol required the use of gloves and protective yellow clothing before entering the VRE area and changing into regular hospital gear before leaving the VRE area. Health professionals (e.g., x-ray technicians) who needed to see patients in both groups were instructed to begin in the clean area and to follow the above protocol in the VRE area.
7. All parents were informed by the attending physicians in the NICU about the outbreak, and provided with a written explanation. Information was provided to parents and visitors about the requirement of hand hygiene and the reasons for the enhanced infection control measures for VRE-fm colonized babies.
8. A separate entrance, an area for breastfeeding, and a waiting area were designated for families of infants in the VRE group. These families were given special instruction on the importance of hand hygiene and were asked not to enter other areas of the hospital.
9. The education of newborn service and visiting hospital staff now centered around hand hygiene and on the potential role of healthcare workers in the transmission of VRE-fm.
10. The rates of adherence to the WHO guideline on Hand Hygiene [15] were monitored and included in the feedback to the units throughout the outbreak period.

### Sample collection, screening and antibiotic susceptibility evaluation

Stool swabs were collected from patients, inoculated on CHROMagar VRE™ plates (Hylabs, Rehovot, Israel) and incubated at 36 °C for 18–24 h. Growth of colonies was detected according to the manufacturer’s instructions, and colony species were identified by the VITEK-MS® system (bioMérieux SA, Marcy l’Etoile, France). Vancomycin resistance and antibiotic susceptibility were determined by VITEK® 2 (bioMérieux SA, Marcy l’Etoile, France).

### Environmental investigation

Environmental sampling was performed in an attempt to identify a possible environmental source for the outbreak. In total, 10 environmental sites were sampled with premoistened wipes (Polywipe®, Medical Wire and Equipment, Wiltshire, UK), including sinks, taps, and incubators. Furthermore, the samples collected in the NICU included handles of hand disinfection dispensers, soap dispensers, and a variety of environmental surfaces. The wipes were inoculated on CHROMagar VRE™ plates (Hylabs, Rehovot, Israel) and then incubated in a brain heart infusion broth (BHI, Hylabs, Rehovot, Israel). Identification of suspected colonies and analysis of antibiotic resistance were performed as described above. VRE-confirmed colonies were tested for the presence of vancomycin-resistant genes, and clonality was determined by molecular genotyping.

### Detection of the *vanA/vanB* vancomycin resistance genes

PCR amplification was performed for detection of the genes as previously described [16] using the primers *vanA* F: 5′-GGGAAAACGACAATTGC-3′, *vanA* R: 5′-GTACAATGCGGCCGTTA-3′, and *vanB* F: 5′-ACGGAATGGGAAGCCGA-3′, *vanB* R: 5′-TGCACCCGATTTCGTTC-3′ with 68 °C annealing temperature and 35 cycles. *E. faecium* 5842 and *E. faecalis* 3528 harboring *vanA and vanB,* respectively, were used as positive controls.

### Molecular genotyping

Genotyping of VRE isolates was performed using by BOX-PCR. Prior to analysis, each VRE isolate was streaked onto a Mueller-Hinton (MH) agar plate (Hylabs, Rehovot, Israel) and incubated at 37 °C. BOX-PCR was performed as previously described [17] with the following modifications. 100 ng of DNA extracted from one colony from each plate was suspended in 7.5 μl sterile ddH2O and added to 10 μl of PCR mastermix (One*Taq*®, NEB, UK). 0.5 μl (50 μM) BOX A2R primer was added to obtain a final volume of 18 μl. Samples were denatured at 95 °C for 7 min, amplified in 35 cycles of 90 °C for 30 s, 40 °C for 1 min, and 68 °C for 8 min, followed by a final extension step of 68 °C for 16 min. Banding patterns were visualized using QIAxcel ScreenGel software (Qiagen, USA) combined with the QIAxcel Advanced capillary electrophoresis system (Qiagen, USA). A *E. faecium* 5842 reference strain was included as a discriminatory control. Fingerprint patterns of the isolates generated by BOX-PCR were analyzed with GelCompar II software (Applied Maths, Belgium). Dendrograms were created using a densitometric curve-based algorithm (Dice correlation coefficient) and UPGMA to cluster patterns by similarity.

## Results

A total of 49 newborns in the TASMC NICU were identified as having been colonized with VRE-fm during the outbreak. All of the isolates presented the same phenotype of high-level resistance to vancomycin (vancomycin MIC ≥32 μg/ml). All of the isolates were also resistant to ampicillin, clindamycin, and erythromycin, and displayed high-level resistance to gentamicin and streptomycin, but remained susceptible to linezolid. All the isolates harboring vanA gene in. and negative for the vanB gene. Clonality was determined for 37 of the 49 VRE-fm isolates by BOX-PCR. A major clone was identified suggesting that the outbreak was monoclonal. There were no cases of clinical infection.

The index case in this outbreak was a newborn who was transferred from the TASMC NICU for cardiac surgery to another healthcare center’s NICU, in which the policy is to screen transferred patients for drug-resistant organisms including VRE. The newborn screened positive for VRE (asymptomatic carriage) on February 20, 2017, and the director of the other NICU called us immediately to report the results. TASMC NICU policies did not include routine screening for VRE at the time of this outbreak, so that it could not be verified that this had been truly the first case. In an attempt to rule out the possibility of an earlier case, the medical records of the TASMC NICU within the 5 years preceding the outbreak were searched and no clinical VRE infections in the NICU were identified. There was also no record of a newborn having screened positive for VRE at another hospital following transfer from the TASMC NICU.

Environmental monitoring was performed in the NICU in an attempt to identify a possible source of the outbreak. Out of a total of 10 sites, three samples were VRE-fm positive on two different incubators, suggesting that clonal spread might have occurred among patients via the contaminated hands of healthcare workers involved in the care of infected or colonized patients. Isolation of the same epidemic type from environmental cultures also indicated the role of environmental contamination in this outbreak.

**Fig. 1:**
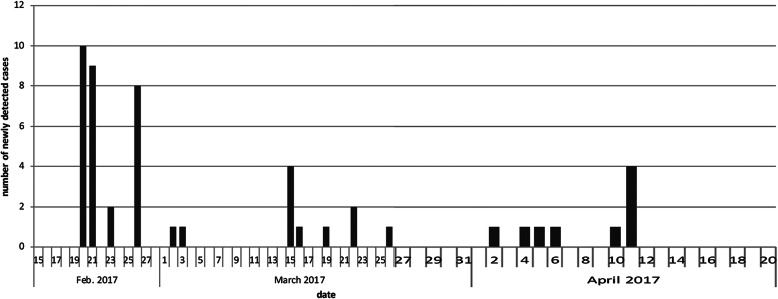
Epidemic curve of VRE outbreak in the NICU. New cases were identified for 7 weeks following the initial NICU screening

After the initial screening of all newborns at the TASMC NICU, 28 additional cases were detected over the following 7 weeks (Fig. 1). Some of these cases had been hospitalized in the clean side of the NICU and were detected by routine repeat screenings conducted in the clean area 3 times a week. Others were exposed infants in the VRE area, who were rescreened at discharge. The rest of the cases had been silently colonized, and there were no clinical infections with VRE. Possible sources of contamination in the clean area were meticulously investigated and more stringent measures were implemented, such as a new policy that required all staff (except the designated clean area staff) entering the clean area to sign in and wear protective clothing before entering. Equipment that entered the NICU from other parts of the hospital (e.g., bassinets) was cleaned thoroughly. Nurses from the epidemiology unit performed daily inspections in the NICU to assess compliance with infection control measures. All regular computer keyboards were replaced by washable keyboards. New admissions to the NICU were limited as follows: (i) out-born transfers were not accepted; (ii) pregnant women hospitalized in the TASMC high-risk antenatal unit who were expected to deliver an infant in need of an NICU admission were offered transfer to another hospital. Every new case of VRE colonization was reported to the district office of the MOH and to the contact nurse at the infant’s health maintenance organization in the community.

The last NICU VRE-fm positive case was detected on April 11, 2017. The VRE area with its designated staff was maintained until the last colonized infant was discharged on July 5, 2017. Screening of all NICU patients for VRE continued twice weekly until July 19th, 2017 (2 weeks after the last case was discharged).

## Discussion

VRE is an epidemiologically important pathogen due to increasing prevalence of antibiotic-resistant strains. Since the pharmacological treatment options are limited, contact precautions should be implemented in VRE colonized and infected patients [18]. This article describes a silent outbreak of VRE-fm in an NICU of a tertiary municipal hospital. The outbreak was initially discovered during routine screening of an infant transferred from the TASMC NICU to another hospital that mandates routine MDROs screenings of all transfer patients. The immediate steps taken by the TASMC staff for containing the outbreak included screening of the entire NICU patient population followed by complete physical separation of carriers and exposed patients from the newly admitted patients. New cases continued to be identified despite the measures that included separation of personnel and families as well as the NICU patients. The greater stringency of the measures included training and monitoring of hand hygiene guideline implementation, as well as meticulous cleaning of equipment brought in from other parts of the medical center. Finally, rather than mere separation into a clean area, new admissions were limited entirely until the outbreak was contained.

Had the index patient not been screened upon transfer by the outside receiving NICU, it is likely that this large VRE outbreak may have gone undetected and uncontrolled until cases of clinical VRE infection had developed like in Ergaz et al. report which describes the utility of the surveillance policy in maintaining a VRE-free environment [7]. Screening can take several forms. Active screening involves prospective sampling of rectal swabs or fecal matter from high-risk patients specifically for the analysis of vancomycin and other antibiotic resistance. Passive screening involves subjecting samples taken for microbiological analysis (such as from surgical wounds) to an additional analysis for antibiotic resistance for clinical purposes, as opposed to prevention purposes. Active screening of at-risk patients has been shown to contribute to VRE prevention, although the exact mechanism has not been verified, and this effect may be secondary to an increased awareness to control measures [19]. Indeed, many hospitals have not incorporated screening recommendations of VRE to their practices, particularly those with a low prevalence of VRE [20]. Nevertheless, screening had been recommended as early as 20 years ago [14], and the importance of its implementation has been emphasized, even in settings not previously recognized as being prone to outbreaks, such as hospitals in non-endemic countries and NICUs [9, 21].

## Conclusion

In conclusion, it is difficult to contain an outbreak when colonization pressure is very high, as in the herein described case. Our study highlights the importance of screening implementation in a NICU setting since this outbreak could have been prevented by active screening of all out-born transfer patients and by having adopted mandatory screening into the NICU’s routine procedures. To achieve complete sealed separation of carriers, full implementation of all aspects of infection control is needed. In response to this outbreak, the TASMC NICU implemented a protocol of screening upon admission of all transfer patients to the NICU for VRE, as well as for CRE and MRSA.

## Data Availability

The datasets used and/or analysed during the current study are available from the corresponding author on reasonable request.

## Abbreviations

TASMC: Tel-Aviv Medical Center
VRE-fm: Vancomycin-resistant *Enterococcus faecium*
NICU: Neonatal intensive-care unit
MDROs: Multi-drug resistant organisms
MoH: Ministry of Health
